# Sequencing and phylogenetic analysis of the complete chloroplast genome of *Arisaema heterophyllum* Blume

**DOI:** 10.1080/23802359.2021.1993460

**Published:** 2021-10-23

**Authors:** Shanyong Yi, Tao Xu, Xiangwen Song, Wei Wang, Wangyang Yu, Bangxing Han

**Affiliations:** a Department of Biological and Pharmaceutical Engineering, West Anhui University, Luʼan, P.R. China; b Anhui Engineering Laboratory for Conservation and Sustainable Utilization of Traditional Chinese Medicine Resources, West Anhui University, Luʼan, P.R. China; c Anhui Qiansouyan Biotechnology Co., Ltd, Luʼan, P.R. China

**Keywords:** *Arisaema heterophyllum*, complete chloroplast genome, phylogenetic analysis

## Abstract

*Arisaema heterophyllum* Blume is a perennial medicinal herb widely distributed in China, Korea and Japan. In this study, the complete chloroplast genome sequence of *A. heterophyllum* was assembled and characterized based on high-throughput sequencing data. The whole chloroplast genome is 170,610 bp in length and contains 95,485 bp large single-copy (LSC) and 22,605 bp small single-copy (SSC) regions separated by a pair of 26,260 bp inverted repeat (IR) regions. It contained a total of 129 genes, including 84 protein-coding genes, 37 tRNA genes, and 8 rRNA genes, with an overall GC content of 34.5%. A phylogenetic tree reconstructed by 30 chloroplast genomes reveals that *A. heterophyllum* is mostly related to the same genus *A. ringens, A. franchetianum* and *A. erubescens.* The complete chloroplast genome of *A. heterophyllum* was the firstly reported and deposited at GenBank under accession number MZ424448.

*Arisaema heterophyllum* Blume is a perennial herbaceous medicinal plant of the *Arisaema* genus of Araceae and widely distributed in China, Korea and Japan (Wang et al. 2018). Its dried tuber is a traditional Chinese medicine with a long history usage, which was named Arisaema and listed in Chinese Pharmacopeia with the function of dissipating binds and dispersing swelling (Wang et al. 2012). Pharmacological analysis indicated that *A. heterophyllum* possessed many pharmacological activities, mainly including anti-tumor (Feng et al. 2016), anti-bacterial (Wang et al. 2004), analgesic (Ye et al. 2010) and anti-infammatory (Wang et al. 2012). Alkaloids, favonoids, plant lectins, lignans and terpenes are its main medicinal ingredients (Yang et al. 2013; Kant et al. 2020). However, its phylogenetic position is not very clear causing on the lack of genomic information. Here, we characterized the complete chloroplast genome sequence of *A. heterophyllum* according to high throughput sequencing technology, which will provide a powerful informatics data for the phylogeny of *A. heterophyllum* and other related species.

The fresh leaves of *A. heterophyllum* were collected from Lu’an, Anhui, China (31°77′N, 115°93′E). Specimens were stored in the Herbarium of West Anhui University (https://hsx.wxc.edu.cn/, Shanyong Yi with the email ysy345283991@163.com) under the voucher number WAU-YYTNX-20210413-1. Total genomic DNA was extracted from the leaves material according to a modified CTAB protocol (Doyle and Doyle 1987). The DNA was stored at −80 °C in our lab. The whole genome sequencing was conducted by Hefei Biodata Biotechnologies Inc. (Hefei, China) on the Illumina Hiseq platform. The filtered sequences were assembled using the program SPAdes assembler 3.10.0 (Bankevich et al. 2012). The DOGMA (Wyman et al. 2004) and BLAST searches were employed for the annotation.

The chloroplast genome of *A. heterophyllum* was determined to comprise a 170,610 bp double stranded, circular DNA (GenBank accession no. MZ424448), which containing two inverted repeat (IR) regions of 26,260 bp, separated by large single-copy (LSC) and small single-copy (SSC) regions of 95,485 bp and 22,605 bp, respectively. The genome was predicted to have 129 genes, including 84 protein-coding genes, 37 tRNA genes, and 8 rRNA genes. Five protein-coding genes, seven tRNA genes and four rRNA genes were duplicated in IR regions. Nineteen genes contained two exons and four genes (*clpP*, *ycf3* and two *rps12*) contained three exons. The overall GC content of *A. heterophyllum* cp genome is 34.5% and the corresponding values in LSC, SSC and IR regions are 32.3%, 27.9% and 41.3%, respectively.

To investigate its taxonomic status, alignment was performed with 30 reported chloroplast genome (full DNA) sequences (*Centella asiatica* and *Sanicula chinensis* were used as outgroup) using MAFFT v7.307 (Katoh and Standley 2013), and a maximum likelihood (ML) tree was produced by FastTree version 2.1.10 (Price 2010). As expected, *A. heterophyllum* is mostly related to the same genus *A. ringens, A. franchetianum* and *A. erubescens* with bootstrap support values of 100% (Figure 1). In addition, the results also showed that the species from the genus *Arisae ma* (*A. heterophyllum*, *A. ringens*, *A. franchetianum* and *A. erubescens*) were closer to those from the genus *Pinellia* (*Pinellia ternata*, *P. peltata* and *P. pedatisecta*) in the genetic relationship among the family Araceae.

**Figure 1.**
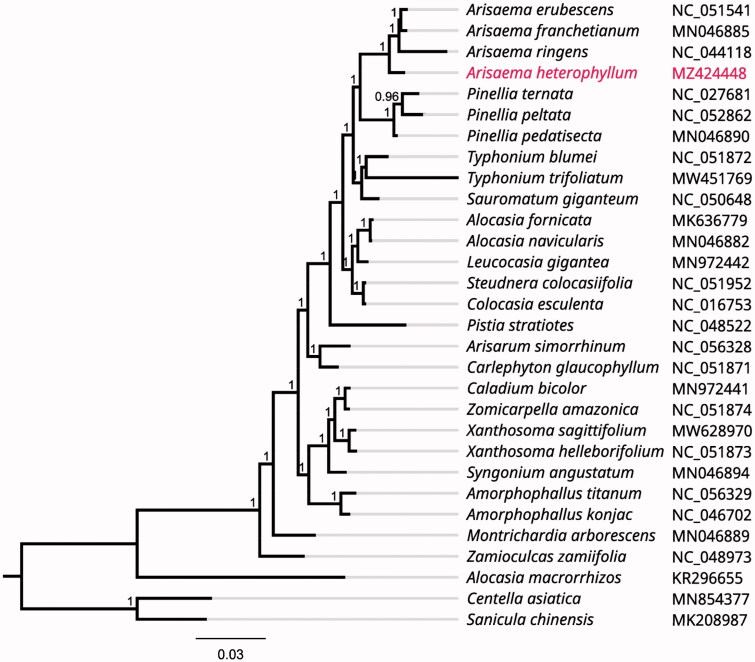
Phylogenetic tree inferred by Maximum Likelihood (ML) method based on 30 representative species. *Centella asiatica* and *Sanicula chinensis* were used as outgroup. A total of 1000 bootstrap replicates were computed and the bootstrap support values are shown at the branches. GenBank accession numbers were shown in Figure 1.

## Data Availability

The genome sequence data of *A. heterophyllum* that support the findings of this study are openly available in GenBank of NCBI at (https://www.ncbi.nlm.nih.gov/) under the accession no. MZ424448. The associated BioProject, SRA, and Bio-Sample numbers are PRJNA743035, SRR15014599, and SAMN19989665, respectively.
